# Wnt/β-catenin signaling pathway: proteins' roles in osteoporosis and cancer diseases and the regulatory effects of natural compounds on osteoporosis

**DOI:** 10.1186/s10020-024-00957-x

**Published:** 2024-10-28

**Authors:** Xiaohao Wang, Zechao Qu, Songchuan Zhao, Lei luo, Liang Yan

**Affiliations:** 1 Department of Spinal Surgery, Honghui Hospital of Xi’an Jiaotong University, Xi’an, China; 2 Xi’an Medical University, Xi’an, China

**Keywords:** Osteoporosis, Osteoblasts, Wnt/β-catenin signaling pathway, Natural compounds

## Abstract

Osteoblasts are mainly derived from mesenchymal stem cells in the bone marrow. These stem cells can differentiate into osteoblasts, which have the functions of secreting bone matrix, promoting bone formation, and participating in bone remodeling. The abnormality of osteoblasts can cause a variety of bone-related diseases, including osteoporosis, delayed fracture healing, and skeletal deformities. In recent years, with the side effects caused by the application of PTH drugs, biphosphonate drugs, and calmodulin drugs, people have carried out more in-depth research on the mechanism of osteoblast differentiation, and are actively looking for natural compounds for the treatment of osteoporosis. The Wnt/β-catenin signaling pathway is considered to be one of the important pathways of osteoblast differentiation, and has become an important target for the treatment of osteoporosis. The Wnt/β-catenin signaling pathway, whether its activation is enhanced or its expression is weakened, will cause a variety of diseases including tumors. This review will summarize the effect of Wnt/β-catenin signaling pathway on osteoblast differentiation and the correlation between the related proteins in the pathway and human diseases. At the same time, the latest research progress of natural compounds targeting Wnt/β-catenin signaling pathway against osteoporosis is summarized.

## Introduction

Osteoporosis is a disease of the skeleton caused mainly by an imbalance in the number or function of osteoblasts and osteoclasts (Delany et al. 2000). Osteoporosis is characterized by decreased bone mass, reduced bone density and thinning of the bone, which makes the bones susceptible to injury and fracture and thus seriously affects the quality of life (Compston et al. 2019). Osteoblasts play an important role in the development of osteoporosis. When osteoporosis occurs, the production and activity of osteoblasts is severely affected, resulting in a decrease in the production or quality of new bone tissue. This causes bones to become weak and easily damaged, increasing the risk of fracture.

The main functions of osteoblasts include promoting bone formation, maintaining bone density and strength, repairing bone damage, and participating in the regulation of bone metabolism (Ponzetti and Rucci 2021). They help keep bones stable and healthy by continuously producing new bone tissue. In the treatment of osteoporosis, promoting the production and activity of osteoblasts can help increase bone density and reduce the risk of fractures, thus improving the symptoms of osteoporosis (Martínez-Reina et al. 2021). Therefore, understanding the differentiation process of osteoblasts is important for the prevention and treatment of osteoporosis.

Osteoblast differentiation is an extremely complex process, involving multiple signaling pathways and regulators such as BMP-Smad and Wnt/β-catenin signaling pathways (Yoshida et al. 2000; Hill et al. 2005). Among them, the Wnt/β-catenin signaling pathway is considered to be one of the most important and critical differentiation pathways, and abnormal activation or inhibition of the Wnt/β-catenin signaling pathway can lead to the development of bone-related diseases, such as osteoporosis and delayed fracture healing (Vlashi et al. 2023). Understanding osteoblast differentiation is crucial for the prevention and treatment of osteoporosis. The study of Wnt/β-catenin signaling pathway not only helps to understand the differentiation mechanism of osteoblasts, but also provides important clues for the treatment of related diseases and develops more effective prevention and treatment strategies. Drugs used to treat osteoporosis include those that inhibit bone resorption, those that promote bone formation, and anabolic drugs (Tonk et al. 2022).However, the associated adverse effects limit their usefulness in treating osteoporosis, and it is urgent to find new therapies for osteoporosis. Meanwhile, in recent years, studies have found that natural compounds have a regulatory effect on the Wnt/β-catenin signaling pathway, showing the ability to regulate bone morphogenesis by affecting the composition or activity of the Wnt/β-catenin signaling pathway. The Wnt/β-catenin signaling pathway is expected to become a therapeutic target. However, the mechanisms by which natural compounds regulate Wnt/β-catenin signaling are not fully understood. Further studies are needed to elucidate the detailed molecular mechanisms and to identify specific compounds with therapeutic potential. Although natural compounds have shown potential to modulate Wnt/β-catenin signaling in laboratory studies, there are still many challenges in translating them into clinical treatments, and natural compounds from different sources may have quality differences that affect the consistency of their therapeutic effects. In view of this, studying how natural compounds regulate the Wnt/β-catenin signaling pathway is not only an important part of understanding the mechanism of bone metabolism and also important for finding new treatments for osteoporosis.

## Wnt/β-catenin signaling pathway

When there is no Wnt signaling or the Wnt receptor is blocked, the Axin-APC-GSK-3-CK1 complex will bind to free β-catenin in the cytoplasm and phosphorylate β-catenin, which will be degraded by the proteasome in the cytoplasm and therefore cannot enter the nucleus (Wiese et al. 2018; Nusse and Clevers 2017). Inside the nucleus, the LEF/TCF transcription factor family binds to the transcriptional repressor Groucho to form a complex protein structure that is unable to act on downstream target genes, leaving the cell surface in a relatively quiescent state(Routledge and Scholpp 2019).

**Fig. 1:**
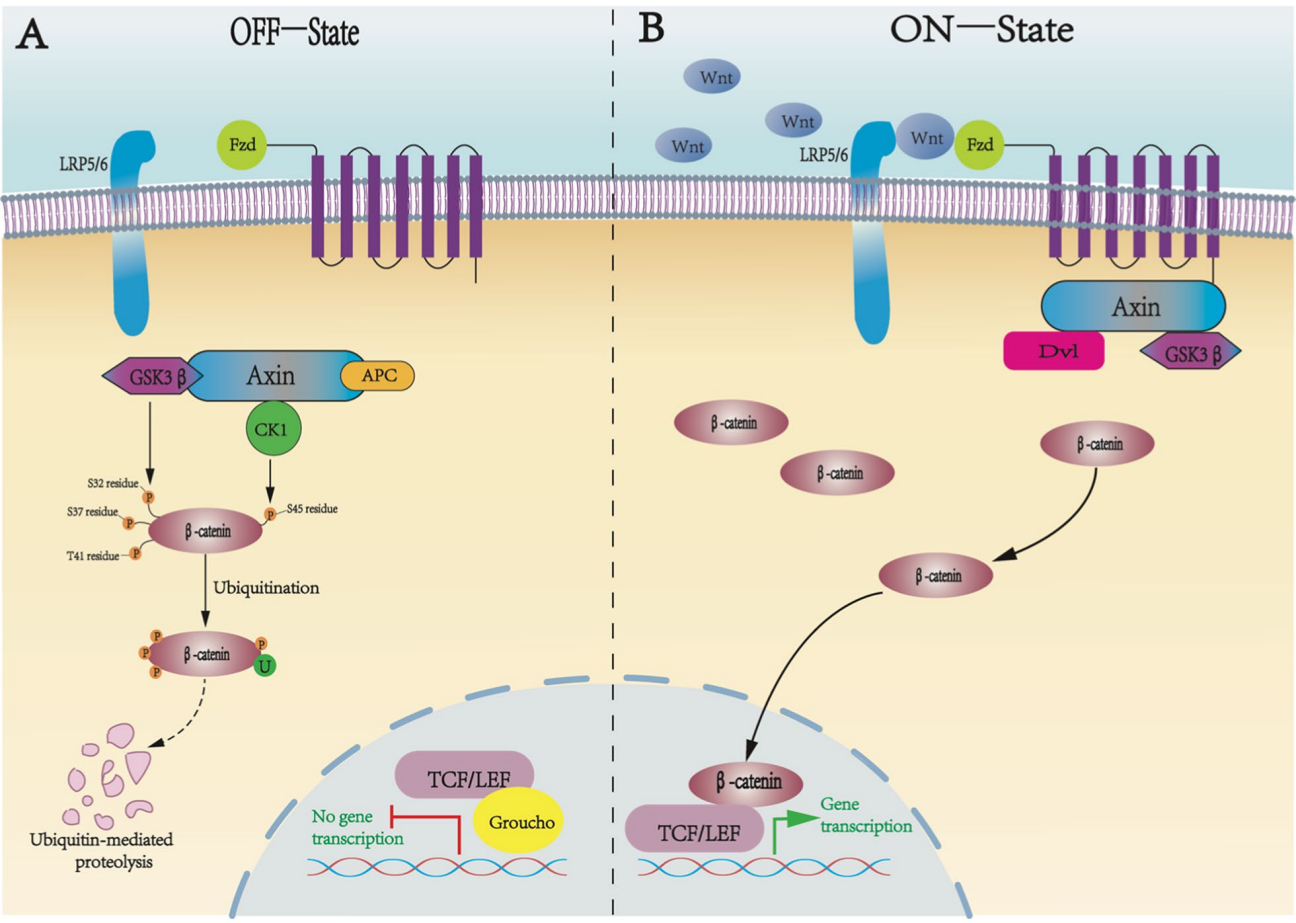
The classic Wnt/β-catenin signaling pathway: mechanisms in closed and open states. **A** In the absence of Wnt ligands, the Wnt/β-catenin signaling pathway is in the off-state (OFF-State). At this time, a disruption complex consisting of GSK3β, Axin, APC, and CK1 phosphorylates β-catenin. The phosphorylated β-catenin is degraded by ubiquitination. In the nucleus, in the absence of β-catenin the LEF/TCF family of transcription factors binds to the transcriptional repressor Groucho and represses the expression of target genes. **B** Wnt /β-catenin signaling is active in the presence of Wnt ligand (ON-State), which binds to LRP5/6, Fed co-receptors, and subsequently recruits Axin, GSK3β, and Dvl, preventing the phosphorylation of β-catenin, and thus preventing the formation of the destruction complex. In the nucleus, β-catenin binds to transcription factors of the LEF/TCF family to promote target gene expression

In the presence of Wnt signaling, Wnt forms a complex with the Fzd receptor and the co-receptor LRP5/6 on the surface of the cell membrane, activating intracellular Dvl proteins and recruiting them to the cell membrane (Xue et al. 2024). The C-terminus of Fzd binds to and phosphorylates the PDZ and DEP regions of the Dvl protein, and the DIX of Dvl interacts with the DIX region of Axin to recruit Axin to the cell membrane, while the kinases GSK-3 and CK1, which bind to Axin, are also translocated to the membrane and phosphorylate the PPPSPxS site at the C-terminal end of LRP5/6, blocking the phosphorylation of β-catenin by GSK-3, and preventing the formation of the Axin-APC-GSK-3-CK1 complex (Bilic et al. 2007). As a result β-catenin cannot be degraded normally and accumulates abnormally in the cytoplasm. A large amount of free β-catenin enters the nucleus to disrupt the complex protein formed by the LEF/TCF family and the transcriptional repressor Groucho, and binds to transcription factors of the LEF/TCF family, while recruiting transcriptional cofactors to further activate the transcription factors, thus activating the expression of downstream target genes (Deldar Abad Paskeh et al. 2021) (Fig. 1).

### Wnt

Wnt proteins are a large family of secreted glycoproteins that play an important role in cell and tissue growth and development (Xiao et al. 2023).Wnt proteins are lipidated in the endoplasmic reticulum by the O-acyltransferase Porcupine (PORCN) while being intracellularly transported and secreted via the membrane protein Wntless (WLS) (Qi et al. 2023). Overall, the mechanism of action of Wnt proteins is divided into two characteristic pathways: (a) the β-catenin-dependent pathway and (b) the β-catenin-independent pathway, which is subdivided into two types, including the planar cell polarity pathway (PCP) and the Wnt/Ca2 + pathway (Ashrafizadeh et al. 2020a, b).Several types of Wnt proteins are known, including Wnt1-19, among which the Wnt1 gene was the first Wnt gene, introduced in 1982 while studying Drosophila melanogaster, and Wnt5a appears to be the major ligand of the non-classical pathway (Ashrafizadeh et al. 2020a, b). However, compared with the β-catenin-independent pathway, the β-catenin-dependent pathway has been studied to a greater extent. These different types of Wnt proteins play different roles in biological growth, development, and energy metabolism. Therefore, further research on Wnt proteins can deepen the understanding of the occurrence and development of diseases, which can guide the treatment of related diseases.

Different types of Wnt proteins play different roles in disease development and treatment. For example, Wnt1 is closely associated with tumor diseases. Milosz Pietrus et al. reported that Wnt1 was associated with the grade and stage of endometrial cancers, with membranous Wnt-1 negatively associated with high-grade endometrial cancers, whereas cytoplasmic Wnt1 was positively correlated (Pietrus et al. 2023). In addition, for cervical cancer, Tie Xu et al. found that overexpression of NEK2 (a serine/threonine kinase involved in mitosis) promotes the development of cervical cancer and affects the sensitivity of cervical cancer to radiation therapy by activating the Wnt/β-catenin signaling pathway through Wnt1 (Xu et al. 2020). On the contrary, Guang-Lin Song et al. found that the microRNA miR-130a-3p (miR- 130a-3p) directly targets Wnt1, and miR-130a-3p can inhibit colorectal cancer(CRC) growth by reducing Wnt1 expression, suggesting a potential microRNA-based therapeutic strategy (Song et al. 2021). The Wnt2 is considered a proto-oncogene protein and is a tumor marker for gastric and CRC, and high expression of Wnt2 predicts a poor prognosis for colorectal and gastric (Liu et al. 2023a, b; Lin et al. 2022). In addition, Wnt2 has also been associated with immunocompetence, and Tu-Xiong Huang et al. showed that Wnt2 secreted by cancer-associated fibroblasts (CAFs) inhibits dendritic cell-mediated antitumour T-cell responses through the SOCS3/p-JAK2/p-STAT3 signaling cascade (Huang et al. 2022).Wnt3 plays a role in embryonic development and neural tube formation. Stephan Niemann et al. found that Wnt3 is closely related to tetra-amelia, a human genetic disorder in which the complete absence of limbs is a major feature (Niemann et al. 2004). In addition, Wnt3 also plays an important role in radiation damage and radiation tolerance. Tong Yuan et al. found that Wnt3 not only promotes the recovery of intestinal stem cells caused by low-dose radiation, but also plays a key role in the inflammatory response and possible death of animals caused by high-dose radiation. This finding reveals that the Wnt3/β-catenin pathway may have dual roles under the influence of different doses of radiation, which can be an important factor in repairing the damage, but also a factor in exacerbating the damage when the radiation dose exceeds a certain threshold (Yuan et al. 2023). Jianbo Liu et al. found that chronic activation of the G protein-coupled estrogen receptor (GPER) promotes the proliferation of intestinal stem cells (ISCc) during menopause, a process that is mediated by the secretion of Wnt3 by paneth cells to activate the Wnt3/β-Catenin signaling pathway, which increases stem cell proliferation (Liu et al. 2023a, b). In addition, HNF4α was found to be a regulator upstream of Wnt3 and paneth cells, a finding that has important implications for the understanding of intestinal health (Jones et al. 2023). In conclusion, Wnt proteins play different and multifaceted roles in different diseases, highlighting the complexity of these signaling molecules. Future studies can further delve into the mechanism of action of different Wnt proteins in disease development, as well as their potential application in clinical therapy, in the expectation that new therapeutic targets can be identified for related diseases and positively impact patient health.

The Wnt-β-catenin signaling pathway plays a crucial role in bone homeostasis by coordinating the differentiation and proliferation of osteoblasts and regulating osteoclast activity. We will focus on the effects of various factors including genetic modifications, environmental pollutants, oxidative stress, mechanical strain and pharmacological interventions on Wnt protein-mediated bone homeostasis. Shu Ma et al. demonstrated that in ankylosing spondylitis, microRNA-96 (miR-96) improves osteoblast differentiation and bone formation through activation of the Wnt signaling pathway, which was achieved by up-regulating the expression of Wnt1, β-catenin, and GSK-3β, as well as induced phosphorylation of β-catenin and GSK-3β activating the Wnt/β- catenin signaling pathway is achieved (Ma et al. 2019). Lu Wu et al. found that cadmium, as a common environmental pollutant, has a serious impact on bone health, especially cadmium chloride (CdCl2), which can reduce the protein levels of Wnt3a, β-catenin, LEF1, and TCF1 and thus inhibit osteogenic differentiation of bone marrow mesenchymal stem cells by suppressing the Wnt/β-catenin signaling pathway (Wu et al. 2019). In addition, the amount of Wnt ligand secreted in the body also affects bone formation. Lawsond et al. showed that mechanical stress promotes the gene expression of Wnt ligands, including Wnt1 and Wnt7b, which enhances the activity of the Wnt/β-catenin signaling pathway and promotes bone formation (Lawson et al. 2022).Notably, Wnt proteins not only promote osteoblast differentiation, but also affects osteoclasts. Osteoclasts also express typical Wnt receptors, and Megan M. Weivoda Ph.D et al. found that Wnt3a can activate β-catenin signaling and thus inhibit osteoclast differentiation, while Wnt3a can also inhibit osteoclast differentiation through activation of cAMP/PKA, which explains that the Wnt protein exhibits a dual regulatory roles in osteoblast and osteoclast differentiation (Weivoda et al. 2016). These findings further emphasize the importance of the Wnt/β-catenin signaling pathway in osteoblast differentiation. further highlight the important role of the Wnt signaling pathway in bone physiology and provide an important theoretical basis for the development of therapeutic strategies to target the Wnt/β-catenin signaling pathway.

### β-catenin

Β-catenin was first discovered as a member of the Adhesion Junction, and was later found to be a core member of the Wnt/β-catenin signaling pathway. β-catenin is an armadillo family protein encoded by the CTNNbl gene, which is rich in the N-terminal Ser and Thr sites and regulates the stability of the molecule (Nichols et al. 2012). The C-terminus consists of 100 amino acids, which activates the transcription of target genes and promotes the initiation and extension of transcription by combining with a series of universal transcription cofactors, such as histone acetyltransferase and chromatin remodeling factor, etc. The intermediate linker arm is divided into 12 Arm repeats (R1 ~ R12), and forms a rod-shaped super-helical structure by the composition of 12 repeats and this structure can effectively prevent proteins from undergoing hydrolysis (Graham et al. 2000). The key to the Wnt/β-catenin signaling pathway lies in the presence of stable β-catenin in the cytoplasm, because β-catenin is an important signaling molecule for the transmission of Wnt signaling to the nucleus (Cheng et al. 2020).

As a key molecule in the Wnt/β-catenin signaling pathway, β-catenin has been widely demonstrated to have an important function in the development of various diseases. Dongying Wang et al. found that the expression of FAT4 was down-regulated in cervical cancer,but FAT4 could bind to β-catenin, prevent it from entering the nucleus, promote β-catenin phosphorylation and degradation, and effectively inhibit the pathological activation of Wnt/β-catenin signaling pathway. In addition, by limiting β-catenin activity, FAT4 is also able to reduce the expression of PD-L1 and STT3A and cause abnormal glycosylation and degradation of PD-L1, which may be beneficial for the enhancement of anti-tumor immune responses (Wang et al. 2023a, b). Hui Hua et al. identified the oncogenic role of lncRNA LINC01226 in gastric cancer, and observed that LINC01226 was able to bind to STIP1 protein, which in turn promoted the dissociation of STIP1-HSP90 complex. This process enhanced the interaction between HSP90 and β-catenin and stabilized the expression level of β-catenin protein, thereby promoting its accumulation in the cell, and as a result, activated the Wnt/β-catenin signaling pathway, which ultimately contributed to the development of gastric cancer (Hua et al. 2023). Ruinan Tian et al. found that the scaffolding protein The receptor for activated C kinase 1 (RACK1) is closely related to tumor development. Proteasome 26S subunit non-ATPase 2 (PSMD2) is a novel binding chaperone of RACK1 and β-catenin. In breast cancer cells, RACK1 prevents ubiquitinated β-catenin from binding to PSMD2 to protect it from proteasomal degradation, thereby stabilizing β-catenin. This promotes the activation of Wnt/β- catenin signaling and the proliferation of cancer cells (Tian et al. 2023). Qing Wu et al. demonstrated that β-catenin plays an important role in the development and progression of diabetes mellitus (DM) and colon cancer. DM induces the shedding of platelet-endothelial cell adhesion molecule 1 (PECAM-1) from endothelial cells and its internalization and aggregation around the nucleus of the cells, which leads to the entry of β-catenin into the nucleus of the cells to promote the progression of colon cancer and endothelial-mesenchymal transition(EndMT) (Wu et al. 2023). Future studies can continue to explore the association between tumors and β-catenin. In addition, it is worthwhile to explore in depth the role of β-catenin in diabetic patients, especially in relation to EndMT. In addition to this, these studies have revealed potential therapeutic targets and strategies, such as using molecules such as NLRP12 and FAT4 to regulate the activation of the β-catenin signaling pathway, which may hold promise for the development of new therapeutic approaches. Future studies could further explore the therapeutic potential of these targets. Overall, these studies have opened up new research directions and therapeutic ideas, and future studies can further deepen the understanding of the role of β-catenin in different diseases and explore the prospects for its application in the clinic.

Recent studies have revealed that β-catenin plays an important role in skeletal physiology and pathology. Skeletal fluorosis, a bone metabolic disease, is characterized by accelerated bone turnover and abnormal osteoblast activity(Srivastava and Flora 2020). Yanru Chu et al. demonstrated that fluoride significantly increased cancellous bone formation and protein expression of Wnt3a, GSK3b, and Runx2 in mice; however, inhibition of β-catenin suppressed the fluoride-induced Runx2 protein expression and osteogenic phenotype, suggesting that β-catenin may be a therapeutic target for skeletal fluorosis (Chu et al. 2020). On the other hand, Sixu Chen et al. demonstrated that the therapeutic effect of PTH is attenuated in mice with type 1 diabetes, but when pre-activated with β-catenin in osteoblasts enhanced the anabolic effect of PTH on bone in mice with type 1 diabetes, significantly improving bone structure, bone volume and bone strength (Chen et al. 2020a, b). In addition, Mark S Rybchyn et al. found that during skeletal mineralization in mice, the complex formed by the binding of calcium-sensing receptor (CaSR) and Homer1 was able to activate AKT via mTORC2 in response to extracellular calcium ion stimulation, thereby stabilizing β-catenin in osteoblasts, a process critical for promoting cell differentiation (Rybchyn et al. 2019). Whereas, Aditi Gupta et al. found that Connexin43 (Cx43), as an intercellular communication protein, can enhance Wnt-dependent and non-Wnt-dependent β-catenin signaling thereby modulating osteoblast function, bone regeneration, and bone metabolism by overlapping its function with β-catenin (Gupta et al. 2019). Microfibrillar-associated protein 5 (MFAP5) is a component of the extracellular matrix genes, which plays a crucial role in the regulation of cell motility and signaling, and Haoran Li et al. found that MFAP5 was able to promote osteoblast differentiation partly through the up-regulation of β-catenin and p-GSK-3β to activate the Wnt/β-catenin signaling pathway (Li et al. 2021). These studies deepen our understanding of the mechanisms of β-catenin's role in skeletal physiology and pathological processes.

### Fzd

The Fzd receptor is a seven-transmembrane protein that is structurally similar to the G protein-coupled receptor. The extracellular N-terminus of the Fzd receptor is a ligand-binding region that binds to Wnt protein, is highly conserved, and is rich in cysteines (Dann et al. 2001). In addition, the intracellular C-terminus is capable of transmitting signals from the extracellular site to the Dvl, which inhibits the degradation of β-catenin and thus regulates the expression of the target gene (Tauriello et al. 2012; Huang and Klein 2004; Punchihewa et al. 2009).

Fzd proteins are associated with a wide range of biological functions and metabolism, thus causing the onset and progression of different diseases when Fed proteins are affected. Ding J et al. developed a specific alternative to Fzd4, L6-F4-2, which improves blood–brain barrier (BBB) or blood-retinal barrier (BRB) function by activating the Norrin/Fzd4/TSPAN12 pathway (Ding et al. 2023). This finding has implications for the treatment of neurodegenerative and ocular diseases involving barrier dysfunction. Zheng S et al. showed that Fzd5 differs from other Fzd proteins in that its conserved extracellular chain region binds cholesterol, which in pancreatic ductal adenocarcinoma (PDAC) promotes the growth of cancer cells through the activation of the Wnt/β-catenin signaling pathway by Fzd5 (Zheng et al. 2022). In addition, Lu P et al. found that deletion of the desert lncRNA HIDEN reduces the interaction between the IMP1 protein and the Fzd5 mRNA thereby leading to destabilization of the Fzd5 mRNA, which is necessary for endodermal differentiation (Lu et al. 2023). Wang L et al. demonstrated that in prostate cancer, GIPC2 can directly bind to Fzd7 through its PDZ structural domain, which activates the Wnt/β-catenin cascade to promote metastasis of prostate cancer cells (Wang et al. 2022a, b). Notably, Fzd6 plays an important role as a key gene in the regulation of tissue polarity, and Dong B et al. demonstrated that knockdown or knockout of Fzd6 in melanoma reduced tumor cell invasion but did not affect proliferation (Dong et al. 2023). These studies have shown that Fzd proteins are involved in signaling pathways that affect a variety of physiological and pathological processes, including prostate cancer, PDAC, and human endodermal differentiation. Therefore, research on Fed proteins can help us to further understand the mechanisms of different diseases and develop new drugs targeting Fed proteins, which can provide new ideas for the treatment of diseases.

Fzd proteins serve as key members of the Wnt/β-catenin signaling pathway, which is critical for osteoblast function. Kushwaha P et al. found that matrix mineralization, expression of osteoblast differentiation markers, and response to Wnt3a were significantly attenuated in osteoblasts deficient in Fzd4, and furthermore, expression of Fzd8 was increased in Fzd4-deficient osteoblasts, which suggests that there may be some degree of functional overlap between Fzd4 and Fzd8 (Kushwaha et al. 2020). In addition, fibroblast growth factor (FGF) is thought to have an important role in osteoblast differentiation, and Ambrosetti D et al. found that genes of the Fzd Wnt receptor family (Fzd1, -2, -7, and -8 genes) that are downregulated by FGF, thereby inhibition osteoblast differentiation (Ambrosetti et al. 2008). In addition, Yu S et al. showed that the transcription factor Specificity protein 1 (Sp1) can regulate the expression of several genes, including those that are essential for growth and developmental processes, and that Sp1 can increase differentiation and mineralization of osteoblasts in vitro through the activation of Fzd1 (Yu et al. 2016). Wang J et al. demonstrated that miR-129-5p targets the Fzd4/β-catenin signaling pathway, inhibits osteoblast differentiation and reduces osteoblast markers (Wang et al. 2021). In conclusion, Fzd plays an important role in bone development and osteoblast differentiation and is influenced by a variety of factors. Therefore, research targeting Fzd may have great therapeutic potential for the treatment of skeletal diseases.

### LRP5/6

LRP5/6 is a low-density lipoprotein receptor-related protein, a co-receptor characterized by a single transmembrane (Jeong and Jho 2021). In addition, it possesses an intracellular region and an extracellular region, each with different functions (Hofbauer et al. 2002). The intracellular region can interact with Axin and GSK-3 proteins due to the possession of PPPS/TPxS/T modules (Zeng et al. 2005; Wu et al. 2009). The extracellular domain can be further divided into the epidermal growth factor structural domain and the low-density lipoprotein receptor structural domain, where epidermal growth factor structural domain binds to Wnt and thus transmits signals(Balemans and Van Hul 2007).

LRP5/6 are key co-receptors in the Wnt/β-catenin signaling pathway that play critical roles in various physiological processes and have been implicated in the pathogenesis of different diseases. Borrell-Pagès M et al. found that the transcriptional regulation of LRP5 is influenced by aggregated low-density lipoprotein (agLDL) and that LRP5 is involved in macrophage lipid uptake and transformation to foam cells, which play a key role in influencing the onset and progression of atherosclerosis (Borrell-Pagès et al. 2011). Meanwhile, Lina Badimon et al. showed that both lipid uptake by macrophages involves two molecules, LRP5 and Protein Convertase Subtilisin/Kexin 9(PCSK9), and, interestingly, that LRP5 is also involved in the mechanism of PCSK9 release (Badimon et al. 2021). Wo D et al. showed that LRP5 deficiency exacerbates myocardial ischemic injury, demonstrating a protective effect of LRP5. Dkk1, an inhibitor of the Wnt/β-catenin pathway, enhances the injury response by inducing endocytosis and degradation of LRP5/6 (Wo et al. 2016). Xuemin He et al. demonstrated that the development of autosomal dominant polycystic kidney disease (ADPKD) is closely associated with the LRP5 variant, which has been observed to be involved in the development of tubular fibrosis through the direct regulation of the TGF-β/Smad signaling pathway (He et al. 2020). Selenoprotein P (SELENOP) is characterized by the presence of selenocysteine, which is mainly secreted by the liver.K Sandeep Prabhu showed that SELENOP is a Wnt signaling regulator, which interacts with Wnt3a and LRP5/6 to influence colorectal tumorigenesis (Prabhu 2023). Summarizing the above findings, it is clear that LRP5/6 play an important role in a variety of diseases. Future studies can further explore the regulatory mechanisms of LRP5/6 and find more targeted therapeutic strategies.

LRP5/6 is an important receptor widely involved in the regulation of skeletal cell function, which is critical for bone development, growth and maintenance of bone density. Ke Yu et al. identified circRNA422 in rat bone marrow mesenchymal stem cells (BMSC), which can promote BMSC proliferation and osteogenic differentiation by regulating the expression of SP7 and LRP5, and has a facilitating effect on the early osseointegration process (Yu et al. 2022). Shahida Moosa et al. found that osteogenesis imperfecta (OI) is one of the disorders associated with LRP, and that variants in LRP5 and LRP6 lead to the development of different types of deformities and disorders such as fracture fragility or oligodontia (Moosa et al. 2019). In contrast, Giulia Leanza et al. found that the Lrp5A214V mutation leads to high bone mass and improves bone microarchitecture and bone strength, as well as peripheral glucose metabolism, and could be an ideal treatment for the high risk of fracture in diabetes (Leanza et al. 2021). In addition, the study by Aimy Sebastian et al. demonstrated an enhanced role of Wnt3a in osteoblast proliferation and early differentiation, emphasizing the importance of LRP6 over LRP5 in mediating Wnt3a signaling (Sebastian et al. 2017). This is confirmed by the results of Yan C Riddle et al., who found that LRP5/6 play an important role in mature osteoblasts, and that the lack of either has an effect on bone density and bone structure, with the effect of LRP6 being more pronounced, suggesting that its role in osteoblasts is more critical than that of LRP5 (Riddle et al. 2013). Future studies can delve into the molecular mechanisms of LRP5 and LRP6 in skeletal cell biology and further reveal their association with bone diseases such as fracture healing and osteoporosis. Meanwhile, based on the in-depth understanding of LRP5/6, therapeutic strategies can be further explored, including the development of drugs and therapeutic approaches targeting their signaling pathways to better address clinical problems related to bone health.

### Axin

Axin is a negative regulator of the Wnt/β-catenin signaling pathway and binds to several members of the Wnt/β-catenin signaling pathway. The N-terminal region is the binding region of APC and Axin, the middle region can bind GSK-3, β-catenin and CK1, and the C-terminal region is the region where Axin forms homo-oligomers, which is also known as the DIX structural domain.Axin contains both Axin1 and Axin2 family members and both are structurally similar (Hsu et al. 1999).Axin1 regulates the phosphorylation and stability of β-catenin by forming complexes with other proteins such as GSK-3β, APC, and CK1 (Koyama-Nasu et al. 2015). Axin2, although its expression pattern and function in cells differ from that of Axin1, plays an essential role in embryonic development, especially in in skeletal development (Hiremath et al. 2022). Recent studies have shown that Axin2 is associated with a variety of diseases and tumors related to the skeletal system.

In recent years, more and more diseases with abnormal expression or mutation of Axin have been identified, further demonstrating the important role of Axin in disease development. Xue-Lian Sun et al. study revealed a compelling cellular competition between neural progenitor cells (NPCs) during brain development that aims to weed out unsuitable cells and thus optimize organogenesis, a process triggered by genetic mosaicism of Axin2 and Trp53 (Sun et al. 2023). Lysophosphatidic acid (LPA) is an endogenous phospholipid that plays a crucial role in the regulation of cellular homeostasis and in the malignant behavior of cancer cells through G-protein-coupled receptors, Hosne Ara et al. found that LPA promotes gastric cancer development and progression, and one of the mechanisms includes LPA's ability to dysregulate energy metabolism through the LPAR2 receptor and Axin2 (Ara et al. 2022).Wenjia Li et al. found that RNF146, a RING-type E3 ubiquitin ligase, was modified by SUMOization, which further promoted its interbinding with Axin, accelerated Axin degradation and enhanced Wnt/β-catenin signaling, a process that contributes to the progression of hepatocellular carcinoma (Li et al. 2023). Sonu Singh et al. showed that knockdown of Axin2 promotes mitochondrial biogenesis and dopaminergic neurogenesis in a rat model of Parkinson's disease, which is achieved through modulation of the Wnt/β-catenin signaling pathway (Singh et al. 2018). Axin is abnormally expressed or mutated in a variety of diseases, further demonstrating its important role in disease occurrence and development. Further studies on the function and regulatory mechanism of Axin and exploration of its specific mode of action in different diseases will help to further reveal the regulatory network of the Wnt/β-catenin signaling pathway and provide a deeper understanding of the elucidation of the mechanism of disease occurrence.

Axin is an important protein that plays a significant role in a variety of cell differentiation and proliferation processes. In the skeletal system, the relationship between Axin and osteoblasts has also received increasing attention. MiR-16-5p is a miRNA that can regulate osteogenic differentiation. Jiaxin Duan et al. demonstrated that miR-16-5p in BMSC-derived extracellular vesicles (EVs) can achieve its function of promoting osteogenic differentiation by suppressing the expression of Axin2, providing a new mechanistic explanation of its important role in osteogenesis (Duan et al. 2023). In addition, Sarocha Suthon et al. showed that SNPrs9921222, an intronic variant of Axin1 associated with bone mineral density (BMD), can regulate the onset and progression of bone diseases in human osteoblasts by regulating Axin1 through GATA4 and estrogen receptor alpha (Erα) binding (Suthon et al. 2022). Further studies by Yoshinori Matsumoto et al. found that deficiency of the E3 ubiquitin ligase RNF146 leads to an increase in the stability of its substrate Axin1, which results in impaired Wnt3a-induced activation of β-catenin and reduced expression of Fgf18 in osteoblasts, which further interferes with the processes of osteoblast proliferation and differentiation (Matsumoto et al. 2017). Future studies can try to reveal the fine regulation mechanism of Axin in skeletal system development and diseases, look for the interactions between new miRNAs or gene polymorphisms and Axin, as well as the mechanism of cross-regulation of Axin with other signaling pathways, which may bring new breakthroughs in the diagnosis and treatment of skeletal system diseases.

### APC

APC proteins with their large relative molecular mass can form functional complexes with Axin and GSK-3, among others, to regulate the protein level of β-catenin in the cytoplasm (Kimelman and Xu 2006). APC possesses both β-catenin and Axin binding domains (Suzuki et al. 2004).

APC is a very important cytoskeletal protein that plays a key role in many biological processes such as cell polarity, signal transduction and cell cycle regulation. APC is strongly associated with the development and occurrence of a variety of diseases includingCRC, gastric cancer, abnormal brain development and cognitive impairment. Jiazhuo He et al. found that H. pylori infection causes DNA damage in gastric stem and progenitor cells. However, DNA damage caused by H. pylori infection was also more severe when the APC gene was inactivated, a finding that suggests a role for APC in the association between H. pylori infection and gastric cancer development (He et al. 2023). Aruna S Jaiswal et al. found that N-Methyl-N'-nitro-N-nitrosoguanidine (MNNG) was able to induce colon cancer cells to enter into an arresting state similar to senescent cells, which was associated with reduced levels of APC proteins, in addition to loss of microtubule organization and reduction of telomeric DNA (Jaiswal et al. 2004). K B Kaplan et al. found that chromosome segregation in cells is closely linked to the APC gene, and that carrying a shortened APC gene results in defective chromosome segregation, and that during mitosis, APC localizes to the ends of micro- tubules embedded in kinetochores and forms a complex with the checkpoint proteins Bub1 and Bub3, while experiments have also demonstrated that APC is a high-affinity substrate for Bub kinase, further proving the important role of APC in mitosis (Kaplan et al. 2001).Yukako Yokota et al. demonstrated that APC have an important role in maintaining the polarity architecture of radial glia during brain development, and that in the absence of APC, radial glia lose their polarity and responsiveness to polarity-maintaining signals, resulting in altered cellular function as well as impaired cortical neuron generation and migration (Yokota et al. 2009). Also, J L Mohn et al. found that deletion of the APC gene in brain neurons leads to multiple comprehensive neurodevelopmental deficits, including learning and memory deficits and autism-like behaviors, and that deletion of APC in the hippocampus leads to increased density of synaptic spines, alterations in synaptic function, and associated changes in molecular modifications and cellular adhesion complexes (Mohn et al. 2014). Future studies could focus on delving deeper into the specific mechanisms of APC in disease development, especially in tumors and the nervous system, and looking for potential therapeutic strategies.

APC proteins play important regulatory roles in several biological processes, especially appearing to be crucial in regulating bone metabolism and osteoblast function. Razvan L Miclea et al. found that APC knockout in mouse MSC-like KS483 cells resulted in upregulation of the Wnt/β-catenin and BMP/Smad signaling pathways but osteogenic differentiation was completely inhibited, but the inhibitory effect of APC deletion on cellular differentiation could be mitigated by increasing the concentration of BMP-7 (Miclea et al. 2011). In addition, Jing Ge et al. found that genistein, a predominant soy isoflavon, can promote osteoblast differentiation through activation of the Wnt/β-catenin signaling pathway by the mechanism that genistein by facilitating the activation of autophagy via the transcription factor EB (TFEB), which allows for the degradation of APC,emphasizing the potential therapeutic potential of pharmacological means to treat bone diseases (Ge et al. 2023).

### GSK-3

GSK-3 is a serine/threonine kinase, between the two structural domains of the C-terminal end of the α-helix and the N-terminal end of the β-fold is the binding pocket ATP ( a homologue of AMP-NP), and this catalytic region mainly consists of amino acid residues such as Lys85, Thr138, Asp133 and Gin185 (Capurro et al. 2020).In the Wnt/β-catenin signaling pathway, the mechanism by which GSK-3 plays a negative regulatory role involves phosphorylating β-catenin to promote its degradation; whereas in the positive regulation of this signaling pathway, GSK-3 acts by phosphorylating LRP6 to promote the expression of positive regulators (Takahashi-Yanaga 2013).

Diseases associated with GSK-3 are an area of great interest. GSK-3 plays an important role in cell signaling and is involved in the regulation of a variety of biological processes, including cell proliferation, apoptosis, neurodevelopment and metabolic regulation. Jibin Zhou et al. found that GSK-3 in cardiomyocytes plays an important role in cardiac homeostasis and overall survival, and that the absence of GSK-3 in cardiomyocytes leads to disturbances in mitotic categorization, which ultimately leads to dilated cardiomyopathy (Zhou et al. 2016a, b). Alonso Sánchez-Cruz et al. demonstrated that the loss of photoreceptor cells caused by retinitis pigmentosa (RP), a group of inherited neurodegenerative diseases of the retina, could be reduced when the GSK-3 inhibitor VP3.15 was used and that visual function was protected, and that this protection was achieved by reducing the expression of neuroinflammatory markers (Sánchez-Cruz et al. 2018). Andrey Ugolkov et al. demonstrated that two novel small-molecule GSK-3 inhibitors (9-ING-41 and 9-ING-87) were effective in decreasing the viability of breast cancer cells, and that 9-ING-41 also enhanced the efficacy of the chemotherapeutic drug irinotecan (CPT-11) (Ugolkov et al. 2016). Silvia guill-luna et al. highlighted that Tumor budding grade III CRC is associated with elevated GSK-3 expression levels as well as increased PD-L1 expression in tumor cells. Notably, inhibition of GSK-3 reduced tumor budding through necrotic and apoptotic pathways as well as significantly increased activated immune cells to enhance anti-tumor responses compared to PD-L1/PD-1 blockade approaches, suggesting that GSK-3 is involved in the regulation of tumor differentiation in CRC and may be involved in immune evasion mechanisms (Guil-Luna et al. 2023).

GSK-3, as a signal-regulating molecule, plays an important regulatory role in osteoblasts and osteoclasts. An in-depth study of its role in osteoblasts and osteoclasts is conducive to a better understanding of the mechanisms of metabolic regulation of bone, and provides new ideas and approaches for the treatment of related diseases. Alessandra Gambardella et al. found that the use of the GSK-3 inhibitor AR28 when used allowed endogenous mesenchymal progenitor cells with osteogenic and adipogenic potential to differentiate towards osteogenesis, but that this promotion of differentiation came at the expense of a certain amount of adipogenicity (Gambardella et al. 2011). Masaki Arioka et al. found that osteoclast differentiation was inhibited while osteoblast differentiation was enhanced when Li2CO3 was used locally as a GSK-3 inhibitor, revealing the potential application of lithium as a GSK-3 inhibitor in the treatment of bone injuries (Arioka et al. 2014). These findings provide new ideas and avenues for understanding the mechanisms of metabolic regulation of bone and the treatment of related diseases.

### CK1

CK1 was one of the first protein kinases found to have serine/threonine protein activity and is involved in the regulation of cellular signaling as well as gene expression (Knippschild et al. 2005).It is widely distributed in eukaryotes, and seven isoforms have been identified in mammals, including α, β, γ1, γ2, γ3, δ, and ε (Cheong and Virshup 2011). The N-terminal end of CK1 is a highly conserved structural domain of the kinase, which consists of 290 amino acid residues, with a large variation in the C-terminal end, which varies in length from 40 to 180 amino acids (Knippschild et al. 2005).

With the increasing research on CK1, a number of diseases associated with CK1 have been identified, and the role of CK1 in the development of these diseases has been gradually revealed. Maureen Spit et al. showed that the environmentally independent self-renewal of cancer cells is driven by truncating mutations in RNF43, which prevent the degradation of β-catenin by immobilizing CK1 at the cell membrane, leading to the sustained activation of transcription of target genes, suggesting that truncating mutations in RNF43 play an important role in the development of cancer (Spit et al. 2020). Marilena Carrino et al. found that the survival of multiple myeloma (MM) cells is also closely related to CK1, and that by inhibiting CK1 activity or lowering the level of CK1, autophagic activity and promoting apoptosis in multiple myeloma cells can be reduced, thereby inhibiting tumor growth and survival (Carrino et al. 2019).Yumeng Zhang et al. demonstrated that nicotinamide promoted the differentiation of pancreatic progenitor cells by inhibiting CK1 and ROCK, and demonstrated that inhibition of CK1α and CK1ε promoted the differentiation of pancreatic progenitor cells (Zhang et al. 2021).

Regarding the relationship between CK1 and osteoporosis, no directly relevant research results have been found in the literature. However, according to the currently known information, CK1 plays an important role in the Wnt/β-catenin signaling pathway, which is closely related to the processes of cell proliferation and differentiation, etc. Although it is not clear that there is a relationship between CK1 and the pathogenesis of osteoporosis, from the aspect of its biological roles, we can hypothesize that there exists a little bit of a link between CK1 and osteoporosis, so that in the future research can further explore the potential role of CK1 in the pathogenesis of osteoporosis.

### Dvl

Dvl is a periplasmic protein that is ubiquitous in organismal tissue cells and is essential for intracellular transmission of the Wnt/β- catenin signaling pathway. The Dvl protein contains three major structural domains; the N-terminus contains the DIX region consisting of 51 amino acids, the middle portion is the PDZ region, which is a basic sequence consisting of 80–90 amino acids present within more than 50 proteins such as PSD-95 and ZO-1, and the C-terminus contains the DEP region, which is capable of binding to the Dvl, EGL10, and Pleckstrin proteins (Gan et al. 2008).

Dvl protein, as an important signal-regulating protein, plays an important role in the processes of cell development, tissue repair, and disease development, and its aberrant function and regulatory imbalance are closely related to the occurrence of many diseases. Vincent C Pai et al. found that the expression of ASPM (a novel Wnt coactivator) was progressively up-regulated in primary and metastatic prostate cancer and that ASPM interacted with Dvl-3 to inhibit the enzymesome-dependent degradation and to increase the protein stability of Dvl-3, which enhances Wnt-induced β-catenin transcriptional activity thereby contributing to the development of prostate cancer (Pai et al. 2019). Lingling Liu et al. found that mutations in the Dvl gene were present in human patients with neural tube defects and Dandy-Walker malformations, which resulted in blockage of the Wnt/β-catenin signaling pathway, in addition to the finding that only the Dvl2 p.R633W mutation showed more severe malformations in zebrafish embryos when compared to wild-type Dvl2 toxicity, revealing the important role of Dvl mutations in the pathogenesis of human neurological diseases (Liu et al. 2020).

In the skeletal system, Dvl has also been found to play an important role in osteoblast function. The following section will focus on the regulatory role of Dvl in osteoblasts and its importance to the health of the skeletal system. H-Y Kim et al. found that CXXC finger protein 5 (CXXC5) is a negative feedback regulator of the Wnt/β-catenin pathway, which can inhibit osteoblast differentiation and bone formation, and the main mechanism of this is that CXXC5 exerts a negative feedback effect by binding to Dvl (Kim et al. 2015).Fangfang Zhou et al. identified a protein called ubiquitin-specific enzyme 4 (USP4), which inhibits osteoblast differentiation by removing the Lysine-63-linked polyubiquitin chain on Dvl forming an inhibitory effect on Wnt/β-catenin signaling (Zhou et al. 2016a, b) (Table 1).

**Table 1:** Wnt/β-catenin signalling targets and their effects on osteoblasts and osteoclasts

Research target	Alterations in Wnt/β-catenin signaling	Effects on osteogenesis	References
miR-96	Wnt1, β-catenin and GSK-3β	Promotes osteoblast differentiation and bone formation	Ma et al. (2019)
CdCl2	Wnt3a,β-catenin,LEF1 and TCF1	Inhibition of osteoblast differentiation	Wu et al. (2019)
Mechanical pressure	Wnt1 and Wnt7b	Promotes bone formation	Lawson et al. (2022)
Wnt3a	β-catenin	Causes inactivation of NFATc1 and inhibits osteoclast differentiation	Weivoda et al. (2016)
Fluoride	Wnt3a, GSK3b and Runx2	Increasescancellous bone formation in mice	Chu et al. (2020)
CaSR and Homer1 complexes	β-catenin	Promote osteoblast differentiation	Rybchyn et al. (2019)
Cx43	β-catenin	Regulation of osteoblast function, bone regeneration and bone metabolism	Gupta et al. (2019)
MFAP5	β-catenin and GSK3b	Promotes osteoblast differentiation	Li et al. (2021)
FGF	Fzd1, -2, -7, and -8	Inhibition osteoblast differentiation	Ambrosetti et al. (2008)
Sp1	Fzd1	Increase differentiation and mineralization of osteoblasts in vitro	Yu et al. (2016)
miR-129-5p	Fzd4 and β-catenin	Inhibits osteoblast differentiation	Wang et al. (2021)
circRNA422	LRP5	Promotes osteoblast differentiation and facilitates osseointegration	Yu et al. (2022)
Lrp5A214V	LRP5	Improves bone microarchitecture and bone strength	Leanza et al. (2021)
MiR-16-5p	Axin2	Promotes osteogenic differentiation	Duan et al. (2023)
SNP rs9921222	Axin1	Regulate the onset and progression of bone diseases in human osteoblasts	Suthon et al. (2022)
RNF146	Wnt3a, β-catenin and Axin1	Inhibition of osteoblast proliferation and differentiation	Matsumoto et al. (2017)
Genistein	APC	Promote osteoblast differentiation	Ge et al. (2023)
AR28	GSK-3	Promoting differentiation of endogenous mesenchymal progenitor cells towards osteogenesis	Gambardella et al. (2011)
Li2CO3	GSK-3	Osteoclast differentiation is inhibited while osteoblast differentiation is enhanced	Arioka et al. (2014)
CXXC5	Dvl	Inhibits osteoblast differentiation and bone formation	Kim et al. (2015)
USP4	Dvl	Inhibits osteoblast differentiation	Zhou et al. (2016a, b)

## Natural compound

**Fig. 2:**
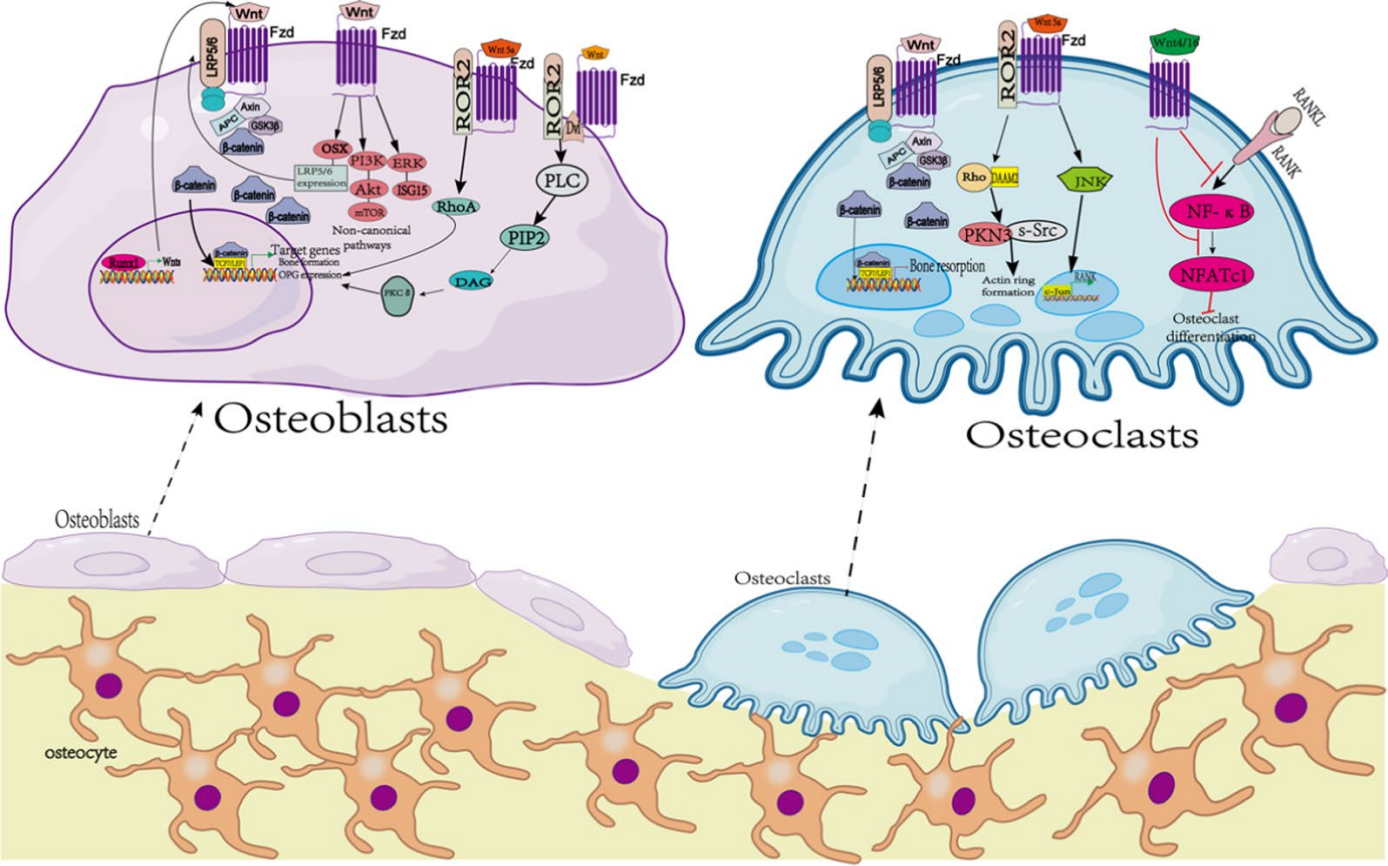
Schematic diagram of Wnt signalling regulation of osteoblasts and osteoclasts. In osteoblasts, β-catenin-dependent classical signalling induces bone formation by promoting osteoblastogenesis and upregulating OPG expression. Non-β-catenin-dependent atypical signalling enhances LRP5/6 expression and promotes osteoblast differentiation. runx1 activates Wnt signalling by increasing Wnts expression and promotes osteoblast differentiation. Non-canonical Wnt5a signalling via ROR2 activates RhoA, which is necessary and sufficient for osteoblast differentiation. In addition, Wnts also promote osteoblast differentiation and bone formation through PLC/PKCδ signalling. In osteoblasts, classical Wnt signalling inhibits bone resorption, while non-classical Wnt signalling promotes bone resorption.Wnt5a-ROR2 signalling enhances RANKL-induced osteoclastogenesis by increasing RANK expression through activation of c-Jun.The Wnt5a-ROR2 signalling pathway activates Rho in an adaptor-dependent manner with the aptamer protein Daam2.Subsequently, the Rho effector kinase Pkn2 activates Rho. Subsequently, the Rho effector kinase Pkn3 binds to c-Src and promotes actin ring formation, thereby increasing bone resorption. Wnt4 and Wnt16 inhibit RANKL-induced activation of NF-kB and NFATc1 signalling, thereby inhibiting osteoclast differentiation

The Wnt/β-catenin signaling pathway is one of the key mechanisms regulating bone metabolism, which influences osteogenic activity and osteoclastogenesis, and maintains the health and regeneration of bone tissue (Lin et al. 2024). However, for how to precisely modulate the Wnt/β-catenin signaling pathway for the treatment of diseases including osteoporosis, fracture and other diseases related to bone loss is still a major challenge for current research. For compounds from nature, which are increasingly valued due to their potential bioactivity and therapeutic potentials, we will summarize important natural compounds that have been found to interact with the Wnt/β-catenin signaling pathway, which will provide new strategies for the treatment of diseases (Fig. 2).

A variety of different tissues and organs such as the myocardium, liver, thyroid, adrenal glands, white adipose tissue, and central nervous system release a substance called irisin, which is a muscle-derived factor and is released during exercise (Dun et al. 2013; Aydin et al. 2014). The differentiation of osteoblasts decreases under microgravity, but Zhihao Chen et al. showed that irisin promotes the expression of the osteogenic marker genes Alp, ColIα1, and also promotes osteoblasts differentiation under microgravity by increasing the expression of β-catenin (Chen et al. 2020a, b).

Curculigo orchioides, a traditional Chinese medicine, can treat a variety of diseases including bone atrophy, osteoporosis, and numbness of the limbs (He et al. 2019). The main bioactive components of curculigo orchioides include polysaccharides, phenolics, novolacins and terpenoids (Wang et al. 2021). COP70-1 is a bioactive ingredient isolated from curculigo orchioides that promotes osteoblast differentiation.One of the mechanisms is that COP70-1 induces GSK-3β phosphorylation in a concentration-dependent manner thereby increasing the stabilization and accumulation of β-catenin proteins and transferring them to the nucleus, initiating the expression of TCF/LEF-1, which promotes osteogenesis by mediating the Wnt/β-catenin signaling pathway (Wang et al. 2023a, b).

Ginkgolide B (GB) is a bioactive ingredient isolated and purified from Ginkgo biloba. It is widely used for its antitumor and anti-inflammatory activities, Bing Yao et al. has found that GB has an inhibitory effect on prostate cancer (Yao et al. 2017).Bin Zhu et al. have demonstrated that GB promotes ALP activity and mineralization in osteoblasts, and upregulates genes that are hallmarks of osteoblast differentiation, such as Col I, Runx2, Osterix, OCN, and OPN, and that the mechanism of this is that GB promotes differentiation of osteoblasts by increasing the level of Ser9 phosphorylation of GSK-3β, upregulating the expression of β-catenin, and activating Wnt/β-catenin signaling (Zhu et al. 2019).

Paeonia lactiflora (PL) is often used to treat a variety of conditions such as irregular menstruation and dysmenorrhea, and has a variety of biological activities such as anti-inflammatory, anti-cancer, and anti-pigmentation. Albiflorin, the main active ingredient in peony, is essentially a monoterpene glycoside. Albiflorin is able to prevent osteoporosis through antioxidant action (Suh et al. 2013) and also reduces inflammation in the lungs (Cai et al. 2019) and improves memory deficits (Xu et al. 2019). Recently, Jae-Hyun Kim et al. found that albiflorin can promote osteoblast differentiation and mineralization. Albiflorin increased the protein expression of BMP-2, RUNX2, and OSTERIX as well as the phosphorylation of Smad, which suggests that it can stimulate bone production through the BMP-2/Smad signaling pathway. In addition, it was found to increase the mRNA expression of Wnt10b, β-catenin, LRP5, LRP6, Dvl2, and cyclin D1, which demonstrated that it could stimulate osteoblast differentiation and regeneration through the Wnt/β-catenin signaling pathway (Kim et al. 2021).

3,5-dicaffeoyl‐epi-quinic acid(DCEQA), a substance extracted from atriplex gmelinii, is a derivative of chlorogenic acid and is biologically active. Anti-obesity and UV photoprotective effects of DCEQA have been reported. Recently, Fatih Karadeniz et al. demonstrated that treatment of human bone marrow mesenchymal stromal cells (hBM-MSCs) with DCEQA promotes their differentiation to osteoblasts, as well as enhances ALP activity and cellular mineralization, and one of the main mechanisms of this is that DCEQA increases the levels of Wnt 10a and Axin proteins, as well as phosphorylation of β-catenin, which promotes the Wnt/β-catenin signaling pathway and thus enhances osteoblasts' differentiation (Karadeniz et al. 2020).

Euodia sutchuenensis Dode (ESD) is an ornamental tree species which grows in temperate regions. ESD has a high medicinal value, relieving headaches, gastritis and dermatitis, in addition to the anti-mold activity of the geranylfuranocoumarins in its fruits (Stevenson et al. 2003; Adams et al. 2006). Recently, Jeong-Ha Hwang et al. found that ESD extract may have therapeutic potential for the treatment of osteoporosis. They found that ESD extract was able to enhance the ALP activity and mineralization capacity of osteoblasts, and that the expression of osteoblast differentiation marker genes, such as BMP2, RUNX2, and COL1A1, was increased after treatment with ESD extract, and that the mechanism was that ESD extract increased β-catenin levels in osteoblasts, thereby activating the Wnt/β-catenin signaling pathway (Hwang et al. 2015).

Geraniin is a substance with a wide range of biological activities extracted from phyllanthus amarus, which is essentially a cyclic tannin. Geraniin has a wide range of anti-inflammatory, antioxidant, anti-infectious, anti-tumor, anti-hyperglycemic and anti-hypertensive properties (Boakye et al. 2016; Aayadi et al. 2017; Liu et al. 2016; Ren et al. 2017; Elendran et al. 2015; Patel et al. 2011). Kejia Li et al. demonstrated that Geraniin also has a promotional effect on the differentiation and proliferation of osteoblasts. Geraniin treatment enhanced the expression of a series of proteins related to the Wnt/β-catenin pathway (including β-catenin, Fzd2, LRP6, etc.), while inhibiting the expression of Axin2 (Li et al. 2018). These results suggest that Geraniin may enhance osteoblast function by activating the Wnt/β-catenin pathway.

Astragalus is a Chinese herb with a variety of biological activities including anticancer, anti-inflammatory, and antioxidant. Of interest is that astragalus inhibits bone loss in ovariectomized mice (Xia et al. 2014). Astragaloside I (As-I) is the main active ingredient of Astragalus membranaceus. Xun Cheng et al. found that As-I stimulated osteoblast differentiation, increased ALP activity and its mineralization capacity, and the main mechanism was that As-I increased the expression of β-catenin and Runx2 proteins thereby enhancing the Wnt/β-catenin signaling pathway (Cheng et al. 2016).

Bergamottin (BM), a natural compound, is mainly extracted from a variety of citrus fruits. It has a variety of biological activities, including anti-adipogenesis, antioxidant and anti-cancer, etc. (Jung et al. 2021; Ko et al. 2018). Recently, Xue Wang et al. found that BM was able to regulate the Wnt/β-catenin signaling pathway by increasing the expression of LRP6, Wnt3a and β-catenin as well as decreasing the expression of GSK-3β to promote osteoblasts differentiation attenuating the loss of bone mass and improving bone turnover in OVX-induced osteoporosis in mice (Wang et al. 2022a, b).

Psidium guajava has been found to have significant efficacy in slowing down a number of metabolic diseases such as diabetes, obesity and osteoporosis (Devalaraja et al. 2011).Konica Porwal et al. isolated gallic acid, ursolic acid (UA), 2α-hydroxyursolic acid (2α-UA), β-sitosterol and β-sitosterol-D-glucoside from guava extract (GE). Among them, only UA and 2α-UA enhanced ALP activity and mineralization capacity of osteoblasts, and the main mechanism was that 2α-UA was able to up-regulate the mRNA levels of Runx2 and Wnt3a, whereas UA was able to increase the levels of BMP-2, Col1 and Wnt3a in osteoblasts, thereby enhancing the Wnt/β-catenin signaling pathway (Porwal et al. 2017).

Gigas are a nutrient-rich food. Fermented Oyster Extract (FO) is rich in protein and carbohydrates. In addition, lysine and GABA are the most abundant amino acids in FO. These nutrients may have an important role in human health and bone production. Ilandarage Menu Neelaka Molagoda et al. found that FO promoted osteoblast differentiation and increased the expression of specific marker proteins such as RUNX2, ALP, Col1α1, OCN, OSX, and BMP4, as well as increased osteoblast ALP activity and mineralization capacity. One of the mechanisms is the ability of FO to significantly increase the expression of β-catenin, which activates the Wnt/β-catenin signaling pathway (Molagoda et al. 2019).This is important for promoting osteogenesis and maintaining bone health.

Polygonatum sibiricum polysaccharide (PSP) is a substance extracted from the traditional Chinese medicine Polygonum sibiricum. It has a variety of biological activities, including anti-inflammatory and attenuating amyloid-β-induced neurotoxicity (Zhang et al. 2015). Li Du et al. found that PSP were able to promote the differentiation of bone marrow mesenchymal stem cells to osteoblasts, and enhanced the ALP activity and mineralization capacity of osteoblasts. In addition, PSP increased the expression of osteogenic differentiation genes, including COL I, ALP, Runx2 and OCN. The main mechanism is that PSP increase the expression and activation of β-catenin, promoting the accumulation of β-catenin in the nucleus and subsequently interacting with the transcription factor TCF/LEF to activate the Wnt/β-catenin signaling pathway, thereby promoting osteoblastogenesis and function (Du et al. 2016).

Soybean isoflavones (SI) are a class of natural compounds found in soybeans and belong to the isoflavones group. SI mainly include soy glycosides, soy glycosides and soy brassinas, which are considered phytoestrogens and can mimic the effects of estrogen (Cederroth et al. 2010). Studies have shown that SI have a variety of biological activities including antioxidant, anti-inflammatory, anti-tumor, lipid-lowering and bone density improvement. Fang Yu et al. demonstrated that SI can promote osteoblast differentiation and enhance osteoblast ALP activity and its mineralization capacity, the main mechanism of which is that an increase in the concentration of soy isoflavones can increase the expression level of Wnt3a, Wnt7b, and β-catenin proteins and activate the Wnt3a/β-catenin signaling pathway (Yu et al. 2015).

Arbutin, a natural compound extracted from a variety of plants including bearberry leaves, pears, and marjoram (Lamien-Meda et al. 2009), is a natural hydroquinone derivative. Arbutin has a variety of biological activities, including whitening, anti-inflammatory and protection against oxidative stress (Lim et al. 2009; Wu et al. 2014). Xiangji Man et al. found that arbutin promotes osteoblast differentiation and increases ALP activity and its mineralization capacity, the main mechanism of which is that arbutin significantly increases the protein expression levels of Runx2 and β-catenin, thus promoting osteoblast differentiation through the typical Wnt/β-catenin pathway (Man et al. 2019).

Kirenol is a natural compound isolated from Herba Siegesbeckia, including S. orientalis, S. glabrescens, and S. pubescens, and is a diterpene compound (Xiang et al. 2004). Kirenol has biological activities with antioxidant, anti-inflammatory, anti-allergic, anti-adipogenic and anti-arthritic properties (Xiang et al. 2004; Jiang et al. 2011; Huo et al. 2013). Mi-Bo Kim et al. demonstrated that Kirenol can promote osteoblast differentiation and increase the ALP activity and mineralization capacity of osteoblasts, and one of the main mechanisms is that Kirenol increases the mRNA expression of LRP5, Dvl2, β-catenin, and CCND1 genes, and then up-regulates the activity of Wnt/β-catenin signaling pathway (Kim et al. 2014).

Cajanus cajan (L.) Millsp. is a traditional Chinese herb whose fresh leaves are used to treat a variety of ailments including parasitic diseases, oxidative damage, and cancer (Duker-Eshun et al. 2004). Shan Liu et al. found that Cajanolactone A (CLA), a stilbene extracted from Cajanus cajan (L.) Millsp, has a promotional effect on osteoblast differentiation, and that the main mechanism is that it can significantly upregulate the mRNA levels of Wnt3a, Wnt10b, LRP5, Frizzled 4, β-catenin, Runx2, and Osterix, suggesting that CLA promotes osteoblast differentiation by stimulating Wnt/β-catenin signaling pathway (Liu et al. 2019) (Table 2).

**Table 2:** Effects of natural compounds on the Wnt/β-catenin signaling pathway

Compound	Source	Molecular structure	Dosages	Effects of the Wnt/β-catenin signaling pathway	References
Irisin	Muscle		0、1、5、10 nM	β-catenin	Chen et al. (2020)
GB	Ginkgo		0、5、10、20 µmol/L	GSK- 3β、β-catenin	Zhu et al. (2019)
Albiflorin	PL		0、20、40、60 µg/ml	Wnt10b、β-catenin、LRP5、LRP6、Dvl2	Kim et al. (2021)
DCEQA	Atriplex gmelinii		0、1、5、10 µM	Wnt 10a、Axin、β-catenin	Karadeniz et al. (2020)
Geraniin	Phyllanthus amarus		0、0.01、0.1、1、10 nM	β-catenin、Fzd2、LRP6、axin2	Li et al. (2018)
As-I	Astragalus		0、10、20、40 µM	β-catenin、Runx2	Cheng et al. (2016)
BM	Citrus fruits		0、5、10、20 µM	LRP6、Wnt3a、β-catenin、GSK-3β	Wang et al. (2022a, b)
UA	GE		100 pM	Wnt3a、BMP-2、Col1	Porwal et al. (2017)
2α-UA	GE		100 pM	Wnt3a、Runx2	Porwal et al. (2017)
Arbutin	Bearberry leaves, pears, marjoram		0、10、50、100 µM	Runx2、β-catenin	Man et al. (2019)
Kirenol	Herba Siegesbeckia		0、10、20、40 µM	LRP5、Dvl2、β-catenin、CCND1	Kim et al. (2014)
CLA	Cajanus cajan (L.) Millsp.		0、1、2、4 µM	Wnt3a、Wnt10b、LRP5、Fzd4、β-catenin、Runx2、Osterix	Liu et al. (2019)
COP70-1	Curculigo orchioides	–	0、0.8、1.6、3.2 µM	GSK- 3β、β-catenin	Wang et al. (2023a, b)
ESD Extract	ESD	–	0、1、5 µg/ml	β-catenin	Hwang et al. (2015)
FO	Gigas	–	0、50、100 µg/ml	β-catenin	Molagoda et al. (2019)
PSP	Polygonum sibiricum	–	0、5、10、25、50、100 mg/l	β-catenin	Du et al. (2016)
SI	Soybean	–	0、0.1、0.2、0.3 M	Wnt3a、Wnt7b、β-catenin	Yu et al. (2015)

## Conclusions

With the increasing understanding of the Wnt/β-catenin signaling pathway, it has been found that alterations in different proteins in the pathway can cause disease consequences such as cancer, osteoporosis, and dysplasia. The Wnt/β-catenin signaling pathway is known to play a key role in osteoblast differentiation and bone formation. By regulating this pathway, bone formation can be promoted, bone mineral density can be increased, and fracture risk can be reduced. Targeted therapeutic regimens against the Wnt/β-catenin signaling pathway have proven to be promising options for treating diseases. Clinically, therapeutic strategies targeting this pathway, especially small molecule drugs and natural compounds, have shown promising therapeutic effects, which can provide new treatment options for patients with osteoporosis, improve the quality of life of patients, and reduce the disability and complications caused by osteoporosis. It is expected that further studies will be conducted in the future, and effective treatments centered on the Wnt/β-catenin signaling pathway have been identified.

In cancer treatment, the abnormal activation of Wnt/β-catenin signaling pathway is closely related to the occurrence and development of a variety of cancers. Clarifying the specific mechanism of this pathway in different cancers will help to develop specific targeted therapeutic drugs. By inhibiting the over-activation of this pathway, the proliferation, invasion and metastasis of cancer cells can be prevented and the effect of cancer treatment can be improved. In addition, gene modification and other technologies also provide new ideas and methods for the precision treatment of cancer.

In conclusion, the in-depth study of Wnt/β-catenin signaling pathway provides new direction and hope for the treatment of diseases such as osteoporosis and cancer, which has important clinical application value. Future research should further explore the regulatory mechanism of this pathway, develop safer and more effective treatment methods, and provide strong support for clinical treatment.

## Data Availability

Not applicable.

## Abbreviations

CRC: Colorectal cancer
GPER: G protein-coupled estrogen receptor
ISCc: Intestinal stem cells
miR-96: MicroRNA-96
CdCl2: Cadmium chloride
RACK1: Receptor for activated C kinase 1
DM: Diabetes mellitus
PECAM-1: Platelet-endothelial cell adhesion molecule 1
EndMT: Endothelial-mesenchymal transition
CaSR: Calcium-sensing receptor
Cx43: Connexin43
BBB: Blood–brain barrier
BRB: Blood-retinal barrier
PDAC: Pancreatic ductal adenocarcinoma
FGF: Fibroblast growth factor
Sp1: Specificity protein 1
agLDL: Aggregated low-density lipoprotein
PCSK9: Protein Convertase Subtilisin/Kexin 9
ADPKD: Autosomal dominant polycystic kidney disease
SELENOP: Selenoprotein P
BMSC: Bone marrow mesenchymal stem cells
OI: Osteogenesis imperfecta
NPCs: Neural progenitor cells
LPA: Lysophosphatidic acid
EVs: Extracellular vesicles
BMD: Bone mineral density
MNNG: N-Methyl-N'-nitro-N-nitrosoguanidine
RP: Retinitis pigmentosa
MM: Multiple myeloma
CXXC5: CXXC finger protein 5
USP4: Ubiquitin-specific enzyme 4
GB: Ginkgolide B
PL: Paeonia lactiflora
DCEQA: 3,5-Dicaffeoyl‐epi-quinic acid
ESD: Euodia sutchuenensis Dode
As-I: Astragaloside I
BM: Bergamottin
UA: Ursolic acid
2α-UA: 2α-Hydroxyursolic acid
GE: Guava extract
FO: Fermented oyster extract
PSP: Polygonatum sibiricum polysaccharide
SI: Soybean isoflavones
CLA: Cajanolactone A
MFAP5: Microfibrillar-associated protein 5
PSMD2: Proteasome 26S subunit non-ATPase 2
